# Benchmarking second and third-generation sequencing platforms for microbial metagenomics

**DOI:** 10.1038/s41597-022-01762-z

**Published:** 2022-11-11

**Authors:** Victoria Meslier, Benoit Quinquis, Kévin Da Silva, Florian Plaza Oñate, Nicolas Pons, Hugo Roume, Mircea Podar, Mathieu Almeida

**Affiliations:** 1 Université Paris-Saclay, INRAE, MetaGenoPolis, 78350 Jouy-en-Josas, France; 2 Biosciences Division, Oak Ridge National Laboratory, Oak Ridge, TN 37831 USA

**Keywords:** Data publication and archiving, Metagenomics

## Abstract

Shotgun metagenomic sequencing is a common approach for studying the taxonomic diversity and metabolic potential of complex microbial communities. Current methods primarily use second generation short read sequencing, yet advances in third generation long read technologies provide opportunities to overcome some of the limitations of short read sequencing. Here, we compared seven platforms, encompassing second generation sequencers (Illumina HiSeq 300, MGI DNBSEQ-G400 and DNBSEQ-T7, ThermoFisher Ion GeneStudio S5 and Ion Proton P1) and third generation sequencers (Oxford Nanopore Technologies MinION R9 and Pacific Biosciences Sequel II). We constructed three uneven synthetic microbial communities composed of up to 87 genomic microbial strains DNAs per mock, spanning 29 bacterial and archaeal phyla, and representing the most complex and diverse synthetic communities used for sequencing technology comparisons. Our results demonstrate that third generation sequencing have advantages over second generation platforms in analyzing complex microbial communities, but require careful sequencing library preparation for optimal quantitative metagenomic analysis. Our sequencing data also provides a valuable resource for testing and benchmarking bioinformatics software for metagenomics.

## Abstract (summary)

**Table:** 

Measurement(s)	mock community • Metagenomic profiling
Technology Type(s)	Illumina HiSeq 3000 • ThermoFisher Ion Proton and GeneStudio S5 • MGI DNBseq G400 and T7 • Oxford Nanopore Sequencing • PacBio Sequel System • Genome mapping and assembly
Sample Characteristic - Organism	Synthetic microbial communities • Bacteria • Archaea

## Background & Summary

High throughput metagenomic sequencing has drastically changed our understanding of microbial ecosystems. One of the most popular approach is to use metagenomic sequencing, assembly and binning procedures^1–4^ to investigate the structure, functionalities and ecological interactions of microbial communities with their environment or host^5–9^. Most metagenomic studies rely on second generation sequencing providing billions of short sequences in a single run, with the Illumina sequencing platforms being the most widely used^10^. Improvement of third generation sequencing yields millions of long reads per run but are mostly used for genomic assembly procedures^11–13^, and further benchmarking is required to evaluate their performance for quantitative metagenomic analysis.

**Fig. 1:**
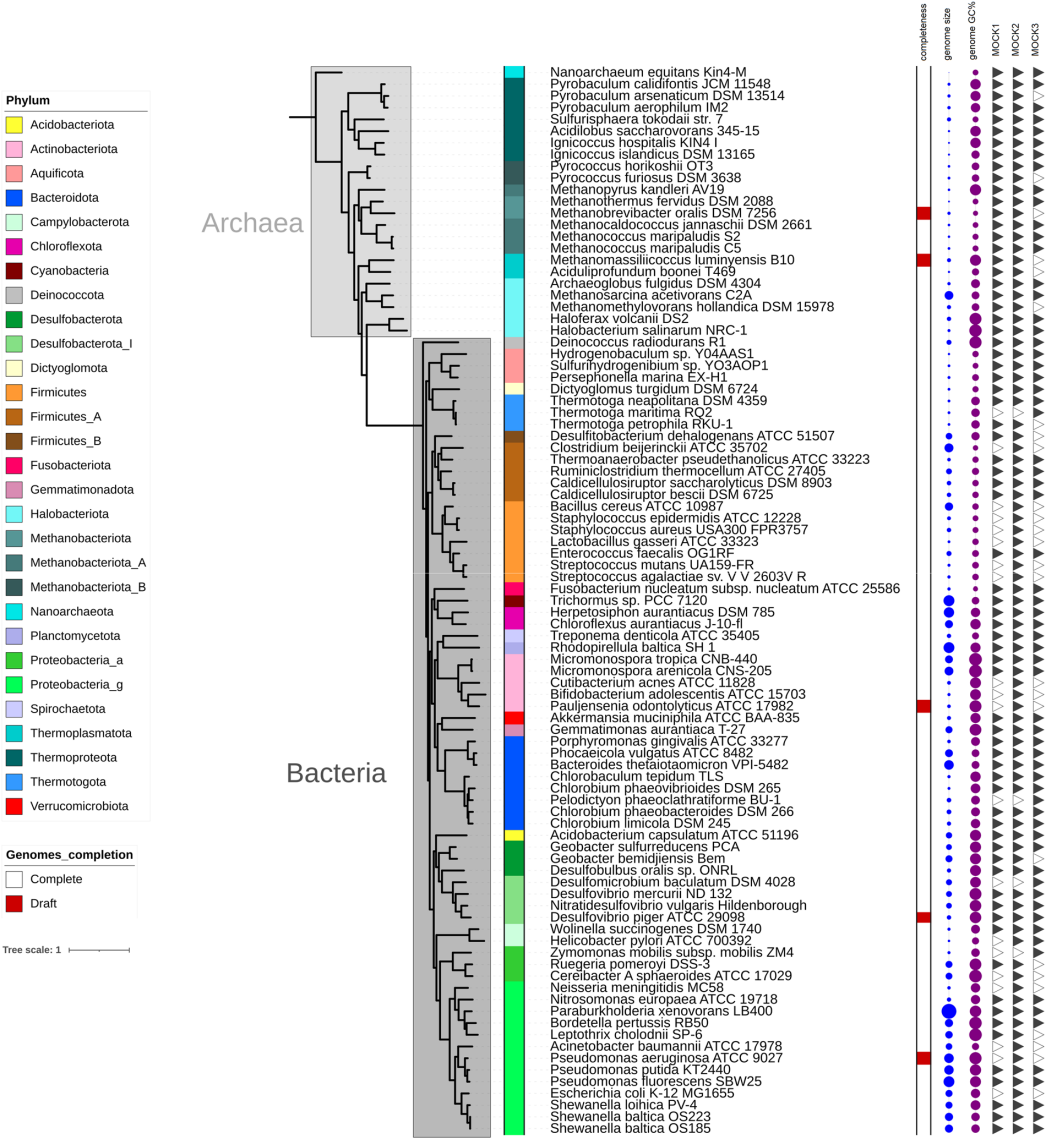
Phylogenetic tree of the microbial species used in the mock microbial communities. Neighbor joining tree built using 40 universal protein markers and visualized using iTOL. On the left, colored strips referred to Phylum phylogenetic ranks using GTBD. Annotations on the right referred to genome completeness (white square, complete; red square, draft genome), genome size, genome GC percent (circle sizes proportional to the dataset range), and mocks composition (plain triangle, present; empty triangle, absent).

For this purpose, we produced three synthetic uneven DNA mocks, varying in their microbial richness (64 to 87 strains, full composition in Supplementary Table S1) and belonging to 29 prokaryotic phyla (Fig. 1). We particularly focused on combining a large spectrum of genome sizes, GC content and mixing closely related species. These mocks represent to date the most complex synthetic communities for evaluating sequencers performances compared to previous studies^14–19^, and not obtained from *in silico* simulated microbial communities^20–23^. We performed five short read sequencing (Ion Proton P1, Ion S5, Illumina HiSeq 3000, DNBSEQ G400, DNBSEQ T7) and two long read sequencing technologies (ONT MinION and PacBio Sequel II), making this study the one covering the widest diversity of sequencing technologies (Table 1).

**Table 1:** Overview of the main sequencing platform characteristics used in this study.

	Illumina HiSeq 3000	Ion Proton P1	Ion S5	DNBSEQ-G400	DNBSEQ- T7	ONT MinION R9	PacBio Sequel II
Amplification type	Solid-Phase bridge	emPCR	emPCR	DNB	DNB	—	—
Sequencing principle	SBS	SBS	SBS	SBS	SBS	SMS	SMS
Average reads length ± stdv after trimming (bp)	149 ± 4.24	144.041 ± 28.43	145.76 ± 28.12	99.91 ± 0.96	99.52 ± 2.58	4408.41 ± 2831.95	10289.7 ± 4036.27
Max read length after trimming (bp)	150	373	347	100	100	60869	40278
SE/PE	PE	SE	SE	PE	PE	SE	SE
Average insert size ± stdv (bp)	433.47 ± 92.37	—	—	245.13 ± 51.04	235.56 ± 54.80	—	—
Total Run Time	4d	4 h	4 h	3d	3d	48 h*	30 h

emPCR = emulsion PCR; SBS: Sequencing by Synthesis; SMS: Single-Molecule Sequencing; DNB: DNA NanoBall. *Data produced by MinION are accessible few minutes after the sequencing start, however, we run the MinION for 48 h to analyze the maximal throughput. DNBSEQ-G400 platform was formerly named MGISEQ-2000. SE: Single end (Forward read only). PE: Paired End. Run Time indicates time to obtain the maximal throughput.

The mock1 (71 strains) was sequenced using all technologies and mocks 2 and 3 (64 and 87 strains, respectively) were additionally sequenced to estimate the impact of various microbial richness (Table 2). After sequencing and quality control, we were able to align more than 99% of all reads for each technology back to their reference genomes, with almost 100% of uniquely mapped reads for long read technologies, down to 87% for Ion Proton and S5 technologies^15,24^. All technologies provided up to 99% identity, except for the MinION R9 with about 89% identity due to a high in/del errors and substitution errors^25^. The PacBio Sequel II provided the lowest substitution error rate and the DNBSeq G400 and T7 the lowest in/dels rate^26,27^.

**Table 2:** Mapping summary per mock and sequencing technology.

Sample ID	Sequencing Technology	N. reads before trimming (million)	N. reads after trimming (million)	%Mapped end-to-end	%Uniquely mapped	%Avg end-to-end best mapped identity	%Avg end-to-end best mapped substitutions	%Avg end-to-end best mapped in/dels
MOCK1 (N = 71 species)	Illumina HiSeq 3000	21.38*2	20.59*2	99.62	93.21	99.45	0.46	0.09
	Ion Proton P1	21.23	20.00	99.29	87.13	99.42	0.12	0.46
	Ion S5	30.05	28.51	99.35	87.13	99.61	0.08	0.31
	ONT Minion R9	0.757	0.696	99.75	99.63	89.08	3.37	7.55
	PacBio Sequel II	0.525	0.524	99.65	99.62	99.72	0.06	0.22
	DNBSEQ-G400	36.17*2	35.42*2	99.22	89.16	99.70	0.30	0.003
	DNBSEQ-T7	404.06*2	375.12*2	98.92	88.78	99.42	0.58	0.003
MOCK2 (N = 87 species)	Illumina HiSeq 3000	24.27*2	23.17*2	99.66	89.44	99.43	0.49	0.08
	Ion Proton P1	22.21	20.98	99.46	86.61	99.41	0.12	0.47
	Ion S5	25.36	24.17	99.55	86.66	99.59	0.09	0.32
	ONT Minion R9	0.919	0.831	99.74	99.61	89.05	3.39	7.56
	DNBSEQ-G400	37.92*2	37.14*2	99.43	88.85	99.73	0.27	0.003
	DNBSEQ-T7	404.99*2	376.04*2	99.03	88.32	99.46	0.54	0.003
MOCK3 (N = 64 species)	Illumina HiSeq 3000	63.97*2	62.08*2	99.58	90.36	99.39	0.52	0.09
	Ion Proton P1	21.50	20.26	99.15	88.33	99.44	0.11	0.45
	Ion S5	27.32	25.90	99.31	88.40	99.62	0.08	0.30
	ONT Minion R9	0.865	0.791	99.79	99.61	89.06	3.35	7.59

The number of reads before and after trimming refer to the sequencing depth (million reads) before and after quality control filtering and trimming. %Mapped end-to-end correspond to the read percentage aligned to a reference genome considering the read full length, while %Uniquely mapped reads correspond to the percentage of reads aligned to only one region of a reference genome. %Avg end-to-end refer to the best hit mean percentage for mapped identity and substitutions and insertions/deletions (in/dels) respectively. See the Method section for trimming and mapping parameters.

**Fig. 2:**
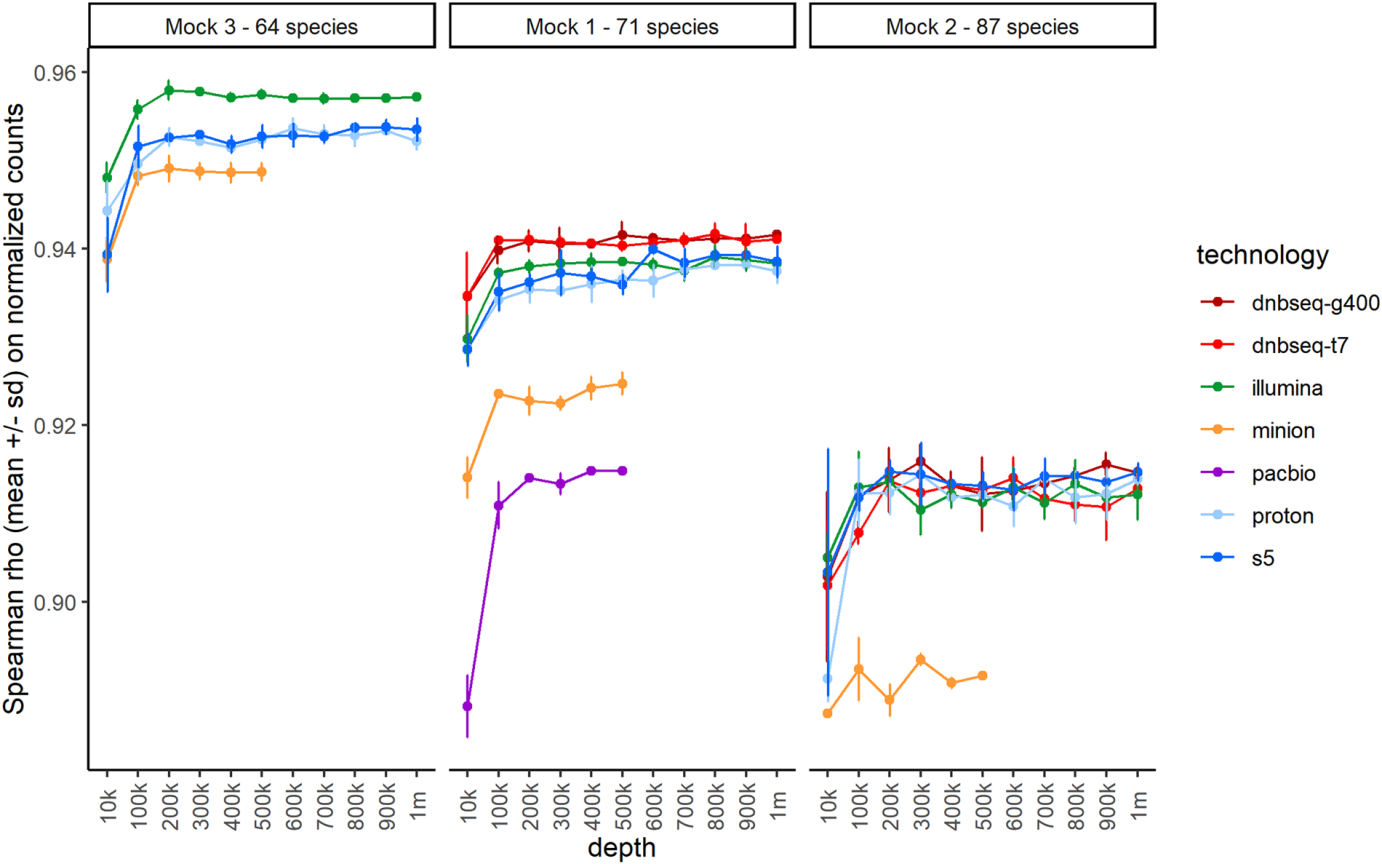
Overall comparison between observed and excepted mock compositions per technology. After read mapping to the mock reference genome, a subsampling was performed 3 times at multiple sequencing depth from 10,000 to 1 million reads, except for ONT MinION and PacBio for which maximum depth was 500k. Spearman rank correlations were calculated between observed genomes abundances normalized by genome size (expressed in %) and the expected mock composition (%). Means ± SD are reported based on the 3 iterations performed per depth. PacBio sequencing was not performed on mock3 and mock2, DNBSEQ-T7 and DNBSEQ-G400 were not performed on mock3.

To evaluate the impact of sequencing depth, we performed a subsampling analysis and compared observed versus theoretical genome abundances (Fig. 2). In general, Spearman correlations were high for all technologies, reaching values above 0.9 when mapping at least 100,000 reads. Notably, correlations were slightly lower for mock communities with higher microbial richness, partially due to cross-matching events during mapping procedures. Whilst second generation sequencers were equivalent for taxonomic profiling^28^, we found more pronounced decreases for MinION and PacBio correlations, even if reads were almost entirely uniquely mapped. Although the PacBio sequencer presented the lowest error rate, these results could be explained by the DNA size filtering step performed during library preparation, which was calibrated to maximize the read length. We hypothesize that the filtering step could remove highly fragmented DNA, thus impacting strains relative abundances^29–31^.

**Fig. 3:**
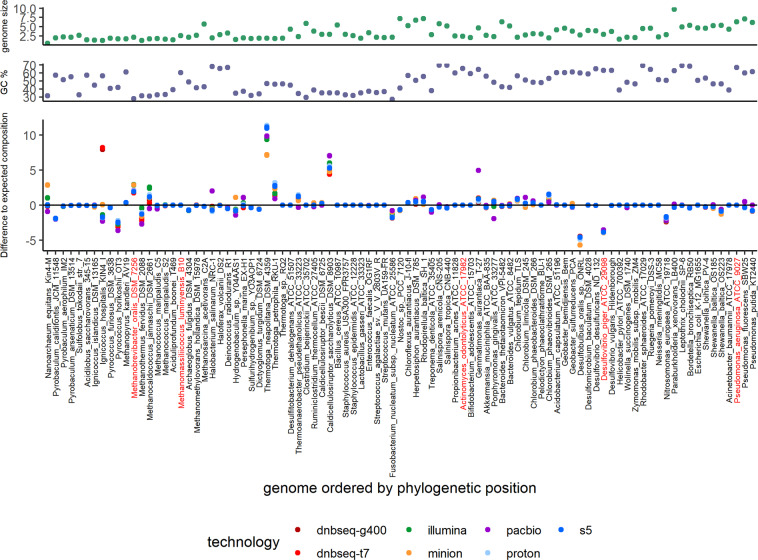
Differential plot between observed and excepted species abundances in mock1. Abundances (%) for each genome were calculated at 500k depth for each sequencing platform and normalized by genome size. Differential abundance was determined by subtracting the excepted abundances (%) to the observed normalized abundances (%). Positive values, genomes are over-estimated; Negative values, under-estimated. Genomes colored in red indicate draft genomes. Genome size and GC percent are reported for each species.

By focusing on mock1 individual genome abundances, we found that most genomes were accurately estimated for all technologies (Fig. 3). Over or under abundance estimation for most genomes was not particularly related to sequencing technology, read length, taxonomy, nor by GC-content, genome size and genome completeness, even at a low depth of 500,000 reads. These results suggest promising opportunities for affordable alternatives to high depth metagenomic sequencing, by using a limited number of reads- the so-called shallow shotgun sequencing- to explore the composition of complex microbiota^32^, even with third generation sequencers^19^.

Finally, we performed *de novo* metagenomic assembly and confronted assemblies with their reference genomes (Table 3). PacBio Sequel II generated the most contiguous assemblies with 36 full genomes out of 71 in mock1, followed by MinION (22 genomes), making third generation sequencers more adapted for genome reconstruction. When considering the mismatches per 100kbps, PacBio Sequel II was also providing the most accurate assemblies, followed by Illumina HiSeq 3000 and DNBSeq G400 (Table 3). However, the lower indels rates obtained with DNBSeq G400 and Illumina HiSeq suggests that hybrid procedures may provide more accurate assemblies than those obtained using long reads alone. We tested this hypothesis by generating hybrid assemblies (Supplementary Information S2) for each technology. For MinION, the hybrid assemblies improved notably the genome fraction recovery compared to MinION assembly only, while reducing the number of fully unaligned contigs, confirming our initial hypothesis. For PacBio, the hybrid assembly did not improve assembly metrics, except for Illumina and DNBSeq with a lower indels rate per 100 kpbs and an improvement in genome fraction recovery with Illumina.

**Table 3:** Mock1 metaquast assembly report.

Sequencer	Ion Proton P1 (spades)	Ion S5 (spades)	Illumina HiSeq 3000 (spades)	DNBSeq G400 (spades)	DNBSeq T7 (spades)	ONT MinION R9 (metaflye)	PacBio Sequel II (metaflye)
Nb Reads (M)	20	20	2 × 10	2 × 10	2 × 10	0.696	0.524
Nb Contigs	45,510	43,879	40,147	44,887	44,603	1,283	**437**
Largest Contig (bp)	384,996	794,907	1,599,668	1,063,396	1,002,925	4,324,150	**7,147,004**
N50 (bp)	7,847	9,089	13,707	8,519	8,184	759,940	**2,013,697**
Genome Fraction(%)	54.767	55.257	61.897	49.397	47.365	44.955	**68.197**
Mismatches per 100kbps	83.29	89.12	47.55	77.22	107.52	339.99	**18.3**
Indels Per 100kbps	77.8	50.03	3.53	3.23	3.67	764.45	11.76
Fully Unaligned Contigs	1,497	1,339	975	735	1,368	231	**6**
Fully Unaligned Length (bp)	900,150	821,545	620,805	426,856	711,992	6,279,694	**134,713**
NB full genome*	5	5	12	7	7	22	**36**

Only contigs > = 500nt were aligned to the mock1 reference genomes. Number of reads, contigs and the size of the largest contig after each assembly are reported, along with: N50 (bp), a common statistic to evaluate the assembly quality; Genome fraction (%), corresponding to the mean percentage of the genome reconstructed during the assembly; the means for Mismtaches per 100kbps and Indels (Insertions/Deletions) per 100kbps, to evaluate the distance of the reconstructed genome to the reference genome). The number of fully unaligned contigs and the respective length (pb) are reported. * Full genome: More than 99% genome recovery.

By this work, we provide a new resource with highly complex synthetic mock samples and extensive metagenomic sequencing data, using the most popular second and third generation sequencing platforms. These data could be used to benchmark or improve metagenomic assemblers, binning software and taxonomic profilers^33,34^.

## Methods

### Synthetic microbial communities’ construction

A total of 91 different strains were used in this study. For 58 strains of Archaea and Bacteria, we used archived gDNA from the Shakya *et al*. study^14^. To further increase the complexity of the constructed community, we cultured nine additional microbes for which the genomic sequence was available. High molecular weight DNA was isolated and quantified as described previously^14^. Purified DNA from 4 other bacteria and archaea was obtained from the laboratory of Dr. Cynthia Gilmour (Smithsonian Research Institute, Edgewater, Maryland, USA). We also used the 20 Strain Even Mix Genomic Material from ATCC (MSA-1002). Three different genomic microbial synthetic communities were assembled by mixing individual, purified DNAs. The composition of each community aimed to provide variation in the number and relative abundance of individual microbe and their represented taxonomic category. The communities achieved a diversity ranging from 64 to 87 strains, representing 29 phyla of Archaea and Bacteria, with a relative abundance distribution spanning over three orders of magnitude. The genome size distribution ranged from 0.49 to 9.7 Mbp and the G + C content was between 27 and 69%. Within the 91 strains, 21 have extrachromosomal DNA such as plasmids or additional chromosomes (Supplementary Table S1). A phylogenetic tree for all strains was constructed using 40 universal protein markers as previously described^1^ and taxonomic ranks were updated using gtdbtk Release 07-RS207^35^. The tree was visualized and annotated using iTOL^36^.

### Library preparation and Sequencing

#### ThermoFisher Ion Proton P1 and Ion GeneStudio S5 library preparation and sequencing

Ion Proton P1 and Ion GeneStudio S5 libraries were built using Ion Plus Fragment Library kit (Thermo Fisher Scientific, Waltham, MD, USA). 500 ng of High Molecular Weight (HMW) DNA was sheared using Covaris E220 sonicator and AFA microtubes (Covaris, Brighton, UK) in 100 µL to achieve maximum distribution at 150pb. After shearing, Ampure XP purification and Qubit quantification were performed. Sheared DNA (100 ng) was submitted to enzymatic treatment steps (End repair, barecode ligation with IonXpress Barcode Adaptors kit and final 9 cycles of PCR amplification). Ampure XP beads were used for size selection to 150 pb after End repair reaction and for purification after the other enzymatic treatment steps. Libraries were quantified and size controlled by using High Sensitivity Small Fragment kit and Fragment Analyzer (Agilent Technologies Inc., Santa Clara, CA, USA). Libraries’ molarity was between 8.000 and 10.000 pM, before normalization at 95 pM and multiplexing for sequencing. Pipetting was performed with Biomek Fxp or Biomek 3000 Liquid handling (Beckman Coulter Inc., Brea, CA, USA). Pre-sequencing step was performed by Ion Chef (Thermo Fisher) for each sequencing device. A first sequencing was performed by Ion Proton with Ion PI HiQ Chef kit (Thermo Fisher) and Ion PI chip kit v3 (Thermo Fisher). Several run were performed including first path, any path with rebalance libraries and final path to get up to 20 million raw reads for each multiplexed sample. A second sequencing was performed by Ion GeneStudio S5 Prime with Ion 550 chef kit and Ion 550 chip kit. A single run was sufficient to obtain up to 20 million raw reads per multiplexed sample.

#### MGI DNBSEQ-G400 and DNBSEQ-T7 library preparation and sequencing

DNBSEQ-G400 and DNBSEQ-T7 libraries were constructed from 500 ng of HMW DNA and fragmented using Covaris sonicator E220 (Covaris, Brighton, UK). Sheared DNA underwent End repair and A-tailing steps as described in the MGI Easy Universal DNA Library Prep Set User Manual v1 (MGI Tech Co., Shenzen, China). Adapters ligation was performed following the instructions of the MGIEasy DNA Adapters kit, and cleaned up with the provided DNA Clean Beads. PCR amplification was carried out on purified adapter-ligated DNA and cleaned-up again using magnetic beads. After quality control using Qubit dsDNA HS Assay Kit (Thermo Fisher Scientific, Waltham, MD, USA), purified PCR products were denaturated and ligated to generate single-strand circular DNA libraries. Barcode libraries were pooled in equal amounts to make DNA Nanoballs (DNB), and sequenced using DNBSEQ-G400 and DNBSEQ-T7 sequencer technologies following the manufacturer’s recommendations.

#### Illumina library preparation and HiSeq 3000 sequencing

DNA libraries have been prepared using the Illumina TruSeq PCR-free HT (Illumina, San Diego, CA, USA), following the manufacturer protocol. Briefly, 2 µg of HMW genomic DNA was fragmented by sonication using Covaris sonicator (Covaris, Brighton, UK). Sheared fragments were cleaned using the Sample Purification Beads provided in the kit, before Ends repair and size selection procedures. Adapters were ligated and libraries underwent an additional cleaning step with magnetic beads. Library quality was assessed using an Advanced Analytical Fragment Analyser (Agilent Technologies Inc., Santa Clara, CA, USA) and libraries were quantified by q-PCR using the Kapa Library Quantification Kit (Illumina, San Diego, CA, US) following the manufacturer’s recommendations. Prior to multiplexing, libraries were normalized to 4 nM and equal volumes were pooled together. Final libraries were sequenced on Illumina HiSeq 3000 using a paired-end read length of 2 × 150 pb with the Illumina HiSeq 3000 Reagent Kits.

#### Oxford Nanopore MinION R9 library preparation and sequencing

Libraries were built with 1D Native barcoding genomic DNA kit (SQK-LSK109 rev E) from Oxford Nanopore Technologies. To increase sequencing yield, 1.5 µg DNA samples were sheared using G-Tube (Covaris) for 2 times 30 seconds at 7,200 rpm. Sheared Fragments (1 µg), of length comprised between 8 and 9 kb, underwent end repair and A-Tailing (New England Biolabs M6630L and E7546L kits). Next, 500 ng of repaired DNA was ligated with adapter barcode using Native Barcoding Expansion 1–12 kit (EXP-NBD104) and Blunt/TA Ligase (New England Biolabs, M0367L). Native barcode ligated DNA was quantified with Qubit and Fragment Analyzer. Equimolar libraries were pooled for a total quantity of 700 ng to ligate to the sequencing adapters. At this step, we choose the Long Fragment Buffer (LFB) from SQK-LSK109 kit to increase the recovery of 3 kb or longer fragments. Pooled libraries were loaded onto R9 FLO-MIN106 flowcell and sequencing was performed during 48 hours.

#### Pacific Biosciences Sequel II library preparation and sequencing

For Pacific Biosciences Sequel II sequencing, 500 ng of HMW genomic DNA was used to make unamplified libraries using the SMRTbell® Express Template Prep Kit 2.0. First, gDNA was sheared to a targeted fragment size of 12 kb using Megaruptor and Long Hydropores (Diagenode, Denville, NJ, USA). Sheared gDNA were concentrated using AMPure PB Beads according to the manufacturer recommendations (Pacific Biosciences, Menlo Park, CA, USA) and underwent two treatment procedures for DNA damage repair and end-repair. Barcoded overhang Hairpins adapters from the manufacturer were ligated to the fragment ends to create SMRTbell templates used for sequencing. SMRTbell templates were purified using an exonuclease procedure to remove any free ends molecules or no adapter templates. Then, size-selection was conducted using Ampure PB beads at a concentration of 0.45X to ensure the removing of short fragments. Our mock SMRTbell template was multiplexed with tree additional samples (not included in this study) to equal molarity. On the resulting template, fragments < 3 kb were removed using an additional diluted Ampure PB beads procedure. PacBio primer v2 annealing to the SMRTbell template and polymerase binding to the annealed template were achieved before being sequenced with Sequel II sequencer using Chemistry 2.0 and 30-hour movie.

#### Sequence QC

The raw reads were quality trimmed using software tools with similar trimming parameters to improve technical comparisons. Illumina HiSeq 3000 and DNBSeq G400 and T7 paired-end reads were trimmed with FASTP v.0.20.0^37^, using Illumina TruSeq adapaters for the Illumina HiSeq 300 sequencer and DNBSeq adapters for the DNBSeq G400 and T7. The minimum read length after trimming was 45nt and all reads with a single N nucleotide or unpaired reads after trimming were discarded. The Ion S5 and Ion Proton reads were trimmed using AlienTrimmer v2.0^38^ by providing the Ion S5 and Proton contaminants and using the following parameters for trimming: “-k 10 -l 45 -m 5 -p 40 -q 20”. The minION R9 reads were base called and quality trimmed using Guppy v2.3.1 + 9514fbc^39^ with the kit SQK-LSK109 and the barcoding kit EXP-NBD103. The PacBio CSS reads were processed through PacBio custom pipeline. Finally, all PacBio and MinION reads shorter than 500nt were discarded.

#### Read mapping procedures

All read mapping procedures were performed on reference genomes corresponding to the expected mock composition. For Illumina, DNBSEQ G400 and T7 platforms, mapping was done with bowtie2 v2.3.5.1^40^ using paired-end best hit end-to-end match and sensitive presets parameters. For Ion Proton and S5, bowtie2 with single-end best-hit end-to-end match and sensitive presets parameters. For MinION and PacBio, mapping was performed with minimap2 version 2.15-r915-dirty^41^ using default parameters, soft clipping activated and by keeping only the best hit.

#### Read subsampling

Subsampling was performed by a python script using the random library and differential analysis in Figs. 2 and 3 between the observed and expected mock composition at different depth, from 10k to 1 M reads were performed under R version 3.6.0 using *stats*, *ggplot2, data.table* and *reshape2* packages.

#### Metagenomic assembly

The Illumina HiSeq 3000, DNBSeq G400 and T7 paired-end reads were assembled with SPAdes v3.14.1^42^ with “--meta” presets and kmer iteration “--k 21,33,55” for DNBSeq, and “--k 21,33,55,77” for Illumina to account for their respective maximal read length. The Ion Proton and Ion S5 single reads were also assembled with SPADES, using the “--iontorrent” and “--careful” flag, as the “--meta” flag is not available for single reads, and a kmer iteration “--k 21,33,55,77”. MinION and Pacbio were assembled with metaFlye v2.8.1-b1688^43^ using the “--meta” preset, “--plasmids” to recover short unassembled plasmids, a minimum overlap of 2000nt, “--pacbio-hifi--hifi-error 0.003” for Pacbio and “--nano-raw” for MinION reads. Finally, the assemblies’ quality was assessed using metaquast v4.6.3^44^.

#### Hybrid metagenomic assembly

Hybrid assemblies were generated using SPAdes v3.14.1^42^ with the same parameters previously described and by adding –pacbio and –nanopore parameters when combining with PacBio reads or MinION reads respectively.

## Data Records

Shotgun metagenomes are publicly available without restriction in the EMBL-EBI European Nucleotide Archive (ENA) under accession number PRJEB52977^45^. All binning and taxonomy assignment results and parameters are available as a publicly shared KBase narrative (https://narrative.kbase.us/narrative/125743) and can also be seen at Figshare^46^.

## Technical Validation

### Library QC checks

Before library preparation by the different sequencing platforms, gDNA mock samples were required to pass quality and quantity controls. Initial DNA quality control included DNA quantification using Quant-iT™ dsDNA Assay Kit broad range (Q33130) reading by FiltermaxF3 (Molecular Devices, Sunnyvale, CA, USA), Qubit dsDNA Assay Kit (Q32853) and fragment analysis using HS Genomic DNA kit (DNG-488-500) on Fragment Analyzer (Agilent Technologies Inc., Santa Clara, CA, USA). The size of initial DNA peak was between 14 and 17 kb without major degradation smear. Additional specific technical validations for DNA integrity were required during each sequencing library preparation to ensure high quality of the final libraries on each platform. Depending on the sequencing technology, these validation steps typically included QC checks after DNA shearing, size selections, purifications on magnetic stands and on the pooled final libraries.

## Usage Notes

Run accession numbers for all metagenomic samples, accessible in the ENA website (PRJEB52977), are fully described in Table 4.

**Table 4:** Shotgun metagenomic datasets description.

	MOCK1 (71 species)	MOCK2 (87 species)	MOCK3 (64 species)
Illumina HiSeq 3000	ERR9765446	ERR9765447	ERR9765448-49
Ion Proton P1	ERR9765780-58	ERR9765759-67	ERR9765768-76
Ion S5	ERR9765777	ERR9765778	ERR9765779
ONT Minion R9	ERR9765780	ERR9765781	ERR9765782
PacBio Sequel II	ERR9765783	NA	NA
DNBSEQ-G400	ERR9765742	ERR9765743	NA
DNBSEQ-T7	ERR9765744	ERR9765745	NA

Run accession numbers were reported for each sample and technology. Metagenomic data have been deposited under BioProject number PRJEB52977.

The protocols and datasets we are presenting in this work can be reused for different applications, in particular to benchmark and improve metagenomic assemblers, taxonomic profilers and binning software. As an example for binning applications, we used three binning software (CONCOCT^47^ v.1.1, MetaBAT2^48^ v1.7 and MaxBin2^49^ v2.2.4) by importing the mock1 Illumina HiSeq 3000 reads, assemblies and hybrid assemblies into KBase^50^ (https://narrative.kbase.us/narrative/125743), with minimum contig size of 1500 nt (Supplementary Table S3). The bins were then optimized using DAS Tool^51^ v1.1.2. We observed comparable number of binned MAGs corresponding to reference genomes using hybrid assembly and higher than using the Illumina short reads dataset alone. With all assemblies and datasets, the recovery of high quality MAGs was not successful for very low abundance genomes, present at less than 0.1% of the mock1 community (Supplementary Table S3).

## Supplementary information

Supplementary Table S1

Supplementary Table S2

Supplementary Table S3

## Data Availability

All reference genomes and scripts for mapping, assembly, genome coverage estimation, subsampling and correlation calculations associated with tables and figures are available at https://forgemia.inra.fr/metagenopolis/benchmark_mock.
